# Topical administration of tranexamic acid reduces postoperative blood loss and inflammatory response in knee arthroscopic arthrolysis: a retrospective comparative study

**DOI:** 10.1186/s12891-023-06349-2

**Published:** 2023-04-05

**Authors:** Junqiao Li, Mingke You, Lei Yao, Weili Fu, Qi Li, Gang Chen, Xin Tang, Jian Li, Yan Xiong

**Affiliations:** Department of Orthopedics, Orthopedic Research Institute, West China Hospital, Sichuan University, Chengdu, 610041 China

**Keywords:** Tranexamic acid, Knee arthrofibrosis, Arthroscopic arthrolysis

## Abstract

**Background:**

Knee arthroscopic arthrolysis serves as an effective treatment for knee arthrofibrosis. However, hemarthrosis is the most common complication in arthroscopic surgery, which has potential adverse effects on postoperative rehabilitation. The purpose of this study was to evaluate the effects of topical tranexamic acid (TXA) in knee arthroscopic arthrolysis.

**Methods:**

A total of 87 patients with knee arthrofibrosis who underwent arthroscopic arthrolysis from September 2019 to June 2021 were eligible for this retrospective review. Patients in the TXA group (n = 47) received topical administration of TXA (50 mL, 10 mg/mL) at the end of the surgery, and patients in the control group (n = 40) received no TXA. The postoperative drainage volumes, hematologic levels, inflammatory marker levels, knee range of motion (ROM), visual analog scale (VAS) pain scores, Lysholm knee scores and complications were compared between the two groups. The curative effect of each group was calculated according to Judet’s criteria.

**Results:**

The mean drainage volumes on postoperative day (POD) 1 and POD 2, and total drainage volume were significantly lower in the TXA group than in the control group (*P* < 0.001 for all). The TXA group had significantly lower postoperative CRP and IL-6 levels on POD 1 and POD 2, and at postoperative week (POW) 1 and POW 2 than the control group. The VAS pain scores in the TXA group were significantly lower on POD 1 and POD 2, and at POW 1 and POW 2 than those in the control group (*P* < 0.001 for all). Patients in the TXA group showed better postoperative ROM and Lysholm knee scores at POW 1 and POW 2. No patient had any complications such as deep venous thrombosis (DVT) or infection. The excellent and good rates of knee arthroscopic arthrolysis were comparable between the two groups at the sixth postoperative month (*P* = 0.536).

**Conclusions:**

Topical administration of TXA in knee arthroscopic arthrolysis can reduce postoperative blood loss and inflammatory response, alleviate early postoperative pain, increase early postoperative knee ROM, and improve early postoperative knee function without increased risks.

## Introduction

Knee arthrofibrosis, defined as the existence of scar tissue in any compartment of the joint resulting in limited range of motion (ROM), is one of the most common complications of knee injury and surgery [1, 2]. It usually occurs after intra-articular injury, long-term external fixation treatment, arthroscopic surgery, total knee arthroplasty and knee infection. Recent studies have shown that 14.5% of patients with knee arthrofibrosis after an intra-articular knee injury undergo surgical treatment. The incidences of knee arthrofibrosis after total knee arthroplasty and anterior cruciate ligament reconstruction (ACLR) were about 5.3% and 4%, respectively [3–5]. However, up to 17% of patients underwent arthroscopic arthrolysis after multiple-ligament reconstruction [6]. At present, there is no uniform standard for the classification of knee arthrofibrosis. According to an international consensus by Kalson et al., the limitation of knee movement was graded as mild, moderate or severe based on the range of flexion (mild: 90° to 100°; moderate: 70° to 89°; severe: < 70°) or extension deficit (mild: 5° to 10°; moderate: 11° to 20°; severe: > 20°) [7]. In addition, Shelbourne et al. classified knee arthrofibrosis into four types according to the loss of extension and flexion and patellar tightness [8]. Conservative treatment options for knee arthrofibrosis mainly include physical therapy and manipulation under anesthesia (MUA). When conservative treatment fails, surgical treatment such as open arthrolysis, quadricepsplasty or arthroscopic arthrolysis is an alternative option [9].

Although the overall complication rate of arthroscopic surgery is relatively low, arthroscopic arthrolysis is not a benign procedure for patients with knee arthrofibrosis [10]. A study including 10,262 arthroscopic surgery cases by Small et al. showed an overall complication rate of 1.68%, among which hemarthrosis was the most common complication, accounting for 60% of the total [11]. Fibrous scar removal and excessive fibrinolysis in lengthy surgery are the leading causes of excessive bleeding after arthrolysis [12]. On the one hand, hemarthrosis can increase postoperative pain and swelling, reduce ROM at the early postoperative stage, and affect rehabilitation training and prognosis [13]. Meanwhile, hemarthrosis has a toxic effect on articular cartilage, thereby increasing the susceptibility to infection and promoting fever [14]. On the other hand, a strong inflammatory response resulting from surgical trauma can lead to postoperative pain and recurrent arthrofibrosis. Therefore, it is crucial for patients with knee arthrofibrosis to reduce postoperative blood loss and inflammatory response in knee arthroscopic arthrolysis.

Tranexamic acid (TXA) is an antifibrinolytic agent composed of a synthetic lysine analogue that stabilizes blood clots and reduces blood loss by competitively inhibiting plasminogen activation [15]. In recent years, TXA has been widely used in orthopedic surgery, including arthroplasty, spine surgery and trauma surgery. The efficacy and safety of TXA in countering the risk of perioperative blood loss have been proven [16]. Several studies have also suggested that the use of TXA in arthroscopic surgery such as ACLR and meniscectomy can reduce postoperative hemarthrosis, swelling and early postoperative pain [17–20]. However, there is currently a lack of studies on the use of TXA in arthroscopic arthrolysis for patients with knee arthrofibrosis. Hence, this study aims to evaluate the effects of topical administration of TXA in knee arthroscopic arthrolysis, including the influence of TXA on postoperative blood loss, inflammatory marker levels, pain, knee ROM and function.

Our hypothesis was that topical administration of TXA would reduce postoperative blood loss and inflammatory response, alleviate early postoperative pain, increase early postoperative knee ROM, and improve early postoperative knee function.

## Materials and methods

### Participants

This study retrospectively reviewed the clinical data of patients with knee arthrofibrosis who underwent arthroscopic arthrolysis in our institution by a senior surgeon from September 2019 to June 2021. Ethical approval was obtained from the Ethics Committee on Biomedical Research, West China Hospital of Sichuan University (No. 2019610). The requirement for informed consent was waived owing to the retrospective nature of the study.

The inclusion criteria were as follows: (1) patients with knee arthrofibrosis caused by knee injury or surgery; (2) patients with a knee extension deficit > 5° and flexion loss > 15°; (3) patients receiving regular conservative treatment ≥ six months before the surgery; (4) patients undergoing knee arthroscopic arthrolysis due to the failure of conservative treatment; and (5) patients with postoperative follow-up ≥ six months.

Patients were excluded from the study if they had (1) incomplete clinical data; (2) a combination of limited ROM caused by extra-articular factors; (3) knee joint ankylosis; (4) knee osteoarthritis of Kellgren-Lawrence grade III or IV; (5) lower limb deformity; (6) previous history of total knee arthroplasty; (7) previous history of knee joint infection or synovitis; (8) simultaneous bilateral procedures; (9) preoperative anemia (Hb < 120 g/L for males and Hb < 110 g/L for females); (10) received perioperative blood transfusion; (11) blood coagulation dysfunction; (12) renal dysfunction; or (13) received anticoagulant or antiplatelet therapy before the surgery.

**Fig. 1:**
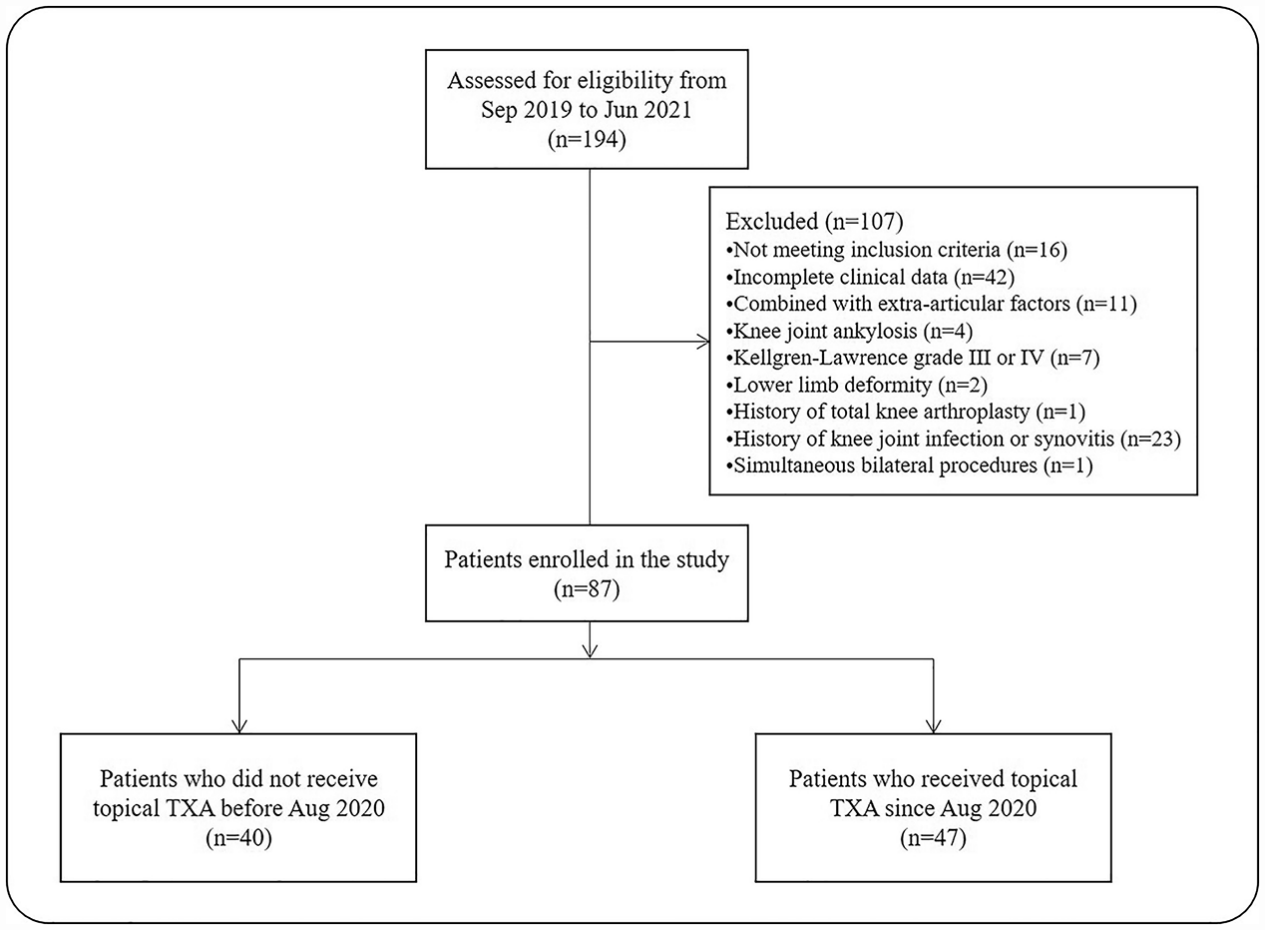
Flow chart of the enrolled patients. *Sep*, September, *Jun* June, *Aug* August, *TXA* Tranexamic acid

A total of 194 patients with knee arthrofibrosis who underwent arthroscopic arthrolysis were initially reviewed, of whom 107 did not meet the eligibility criteria in this study, so 87 eligible patients ultimately remained for data analysis. According to the date of surgery and whether topical TXA was administered, there were 40 patients who underwent knee arthroscopic arthrolysis without topical administration of TXA between September 2019 and July 2020 in the control group, and 47 patients who underwent knee arthroscopic arthrolysis with topical administration of TXA between August 2020 and June 2021 in the TXA group (Fig. 1). The demographic characteristics of the two groups are shown in Table 1. There was no significant difference in age, sex, side, height, weight, body mass index (BMI), cause of knee arthrofibrosis or duration of limitation between the two groups.

**Table 1:** Demographic and perioperative characteristics

Variables	TXA group (n = 47)	Control group (n = 40)	*P*-value
Demographic characteristics			
Age (yr)	40.21 ± 11.14	37.78 ± 8.39	0.259
Sex (male/female)	33/14	31/9	0.442
Side (left/right)	22/25	19/21	0.949
Height (m)	1.68 ± 0.07	1.71 ± 0.09	0.091
Weight (kg)	68.32 ± 12.33	69.83 ± 9.22	0.527
BMI (kg/m^2^)	24.15 ± 3.40	23.88 ± 1.87	0.653
Cause (injury/surgery)	30/17	26/14	0.910
Duration of limitation (mo)	12.74 ± 8.35	15.28 ± 9.98	0.201
Preoperative laboratory values			
Hb (g/L)	147.83 ± 11.95	150.55 ± 10.36	0.264
Hct (%)	44.15 ± 3.17	44.85 ± 2.86	0.285
CRP (mg/L)	2.08 ± 1.07	1.96 ± 0.80	0.565
IL-6 (pg/mL)	2.37 ± 1.34	2.22 ± 0.96	0.936
Preoperative knee condition			
ROM in the clinic (°)	54.89 ± 31.75	56.13 ± 24.66	0.842
ROM under anesthesia (°)	60.64 ± 32.56	61.25 ± 25.03	0.653
VAS pain score	8.09 ± 7.11	7.75 ± 7.33	0.830
Lysholm knee score	62.66 ± 12.35	65.28 ± 13.16	0.342
Surgical data			
Operative time (min)	56.89 ± 13.18	52.93 ± 10.27	0.126
Intraoperative ROM (°)	126.70 ± 10.44	124.63 ± 12.11	0.393

*BMI* Body mass index, *Hb* Hemoglobin, *Hct* Hematocrit, *CRP* C-reactive protein, *IL-6* Interleukin-6, *ROM* Range of motion, *VAS* Visual analog scale.

#### Surgical procedure

The knee arthroscopic arthrolysis was performed under general anesthesia with the patient in the supine position. Before the surgery, passive ROM was assessed in the patient under general anesthesia. A tourniquet with a pressure of 300 mmHg was used after the exsanguination of the affected limb. In the surgery, the standard anterolateral and anteromedial arthroscopic portals were applied. Arthroscopic shaver and radiofrequency ablation were used to remove the hyperplastic fibrous scar tissue in the medial, lateral, intercondylar and suprapatellar compartments. At the same time, intraoperative active bleeding was controlled using electrocoagulation. When necessary, the posteromedial and posterolateral portals were added to remove the posterior fibrous scar tissue and loosen the posterior joint capsule. After completing total intra-articular debridement, continuous and progressive manipulation was performed with the proximal tibia as a lever to achieve the maximal ROM. At the end of the surgery, intra-articular fluid was aspirated, followed by a drainage tube placed in the articular cavity. Patients in the TXA group received an additional topical administration of 50 mL TXA (10 mg/mL; Lummy Inc., China). Then, the drainage tube was clamped immediately, opened three hours after the surgery and removed when the drainage volume fell below 20 mL/day. Meanwhile, the affected knee was compressed with an elastic bandage until extubation. Although the patients in the control group did not receive topical TXA intraoperatively, the rest of the treatment protocol was identical to that in the TXA group.

#### Postoperative rehabilitation

After the surgery, both groups were treated with ice packs to relieve the edema of the affected knee. All the patients received 40 mg q12h parecoxib (Dynastat; Pfizer Inc., USA) intramuscular injection within postoperative 24 hours, with sequential 200 mg bid oral celecoxib (Celebrex; Pfizer Inc., USA) for anti-inflammatory and analgesic therapy for six to eight weeks. Under the guidance of physical therapists, the patients started isometric quadriceps exercises to prevent muscle atrophy and performed ankle pump exercises to prevent deep venous thrombosis (DVT) after recovery from anesthesia. After postoperative 24 hours, all the patients began passive knee flexion exercises using the CPM device three times a day for two weeks to improve knee ROM until the affected knee gradually achieved the maximal ROM. Moreover, the two groups started squatting training and full weight-bearing from the second week after surgery.

#### Clinical outcomes

The primary outcomes were the drainage volumes on postoperative day (POD) 1 and POD 2, and at the time of extubation. The hemoglobin (Hb) and hematocrit (Hct) levels were collected before the surgery, on POD 1, and at the time of extubation. Meanwhile, the levels of C-reactive protein (CRP) and interleukin-6 (IL-6) were detected before the surgery, on POD 1 and POD 2, and at postoperative week (POW) 1, POW 2, postoperative month (POM) 1, POM 3 and POM 6, respectively. The preoperative ROM in the clinic, ROM under anesthesia, intraoperative ROM and postoperative ROM at POW 1, POW 2, POM 1, POM 3 and POM 6 were recorded by a goniometer. Knee function was evaluated by the Lysholm knee score before the surgery and at POW 1, POW 2, POM 1, POM 3 and POM 6. The visual analog scale (VAS) pain score was documented before the surgery, on POD 1 and POD 2, and at POW 1, POW 2, POM 1, POM 3 and POM 6. The operative time, complications and adverse events during the follow-up period were also recorded. Finally, the curative effect of knee arthroscopic arthrolysis in each group was evaluated at the sixth postoperative month.

### Statistical analysis

A post hoc power analysis was performed by G*Power software version 3.1.9.4 (Franz Faul, Universitat Kiel, Germany). According to the literature, a sample size of 87 patients calculated a power greater than 0.9 when the effect size was 0.8 and α = 0.05. The demographic characteristics and clinical outcomes of patients were analyzed using SPSS software version 26.0 (IBM Corp., USA). The quantitative data were presented as the means and standard deviations and compared by the independent Student’s *t*-test or the Mann‒Whitney *U* test. The qualitative data were presented as frequencies or percentages and compared by the Chi-square test. According to Judet’s criteria (excellent: > 100°; good: 80° ~ 100°; fair: 50° ~ 80°; poor: < 50°), the curative effect was calculated using the following formula: excellent and good rate = [n (excellent) + n (good)]/n [21]. *P* < 0.05 was considered statistically significant for all analyses.

## Results

The preoperative laboratory values, preoperative knee condition and surgical data were comparable between the two groups of patients (Table 1).

Table 2 shows the postoperative drainage volumes and hematologic levels in the two groups. The mean postoperative drainage volumes on POD 1 and POD 2, and total drainage volume were significantly lower in the TXA group than in the control group (*P* < 0.001 for all). There were significant differences in the mean hemoglobin levels on POD 1 (*P* = 0.030) and at the time of extubation (*P* < 0.001), and in the mean hemoglobin loss (*P* < 0.001) between the two groups. Correspondingly, the mean hematocrit levels in the TXA group on POD 1 and at the time of extubation were significantly higher than those in the control group with a significantly lower mean hematocrit loss (*P* < 0.001 for all).

**Table 2:** Postoperative drainage volumes and hematologic levels in the two groups

Variables	TXA group (n = 47)	Control group (n = 40)	*P*-value
Drainage volume (mL)			
POD 1	69.26 ± 35.93	153.88 ± 64.02	<0.001
POD 2	36.49 ± 28.85	94.38 ± 48.39	<0.001
Total	113.19 ± 69.62	284.75 ± 129.35	<0.001
Hb (g/L)			
POD 1	140.15 ± 12.79	134.73 ± 9.49	0.030
Extubation	137.04 ± 12.54	128.08 ± 9.83	<0.001
Loss	10.79 ± 4.32	22.48 ± 6.02	<0.001
Hct (%)			
POD 1	41.19 ± 3.35	37.93 ± 3.24	<0.001
Extubation	40.06 ± 3.60	35.38 ± 3.06	<0.001
Loss	4.15 ± 1.76	9.48 ± 2.10	<0.001

*POD* Postoperative day, *Hb* Hemoglobin, *Hct* Hematocrit, *CRP* C-reactive protein, *IL-6* interleukin-6.

**Fig. 2:**
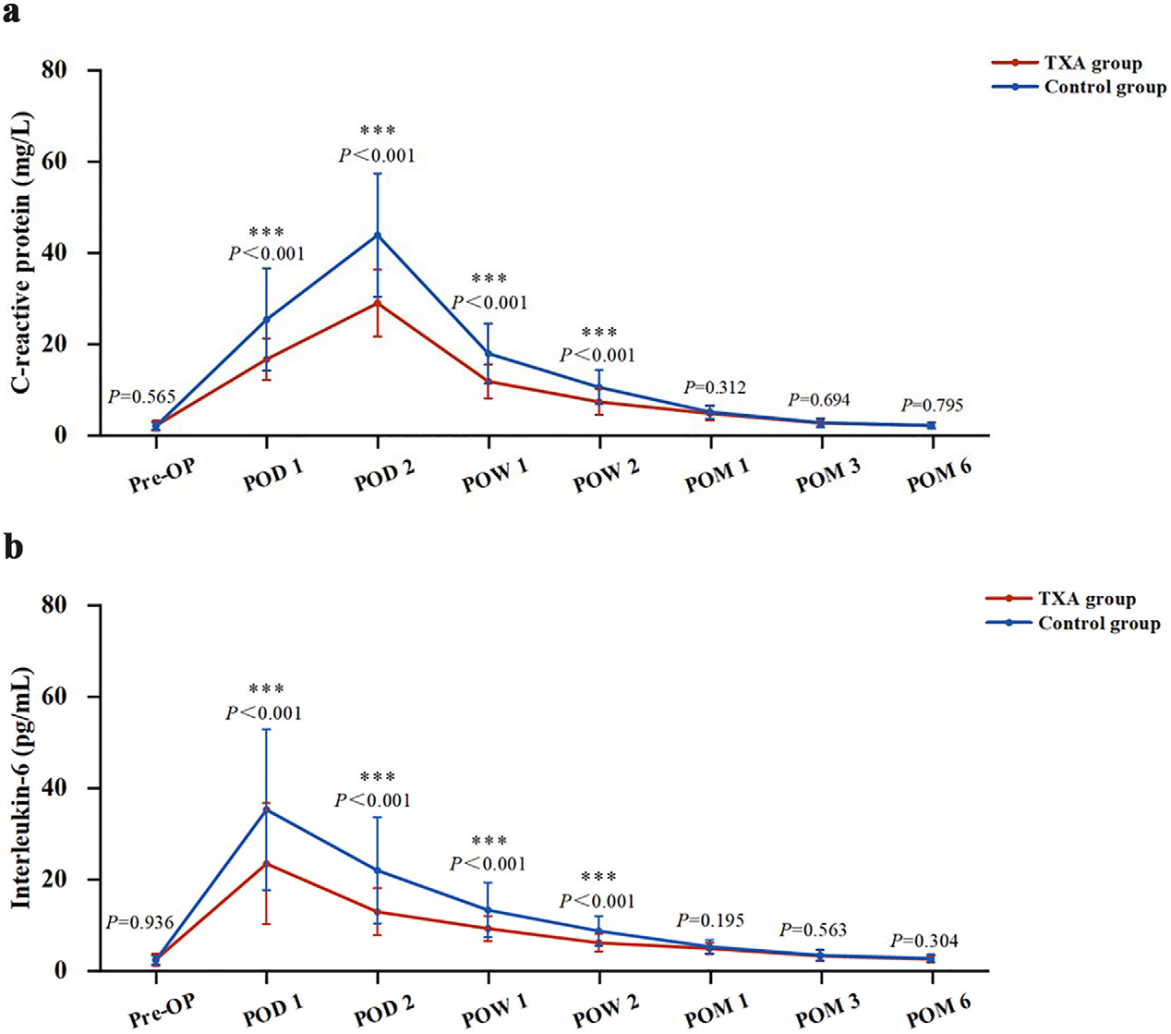
Perioperative C-reactive protein levels (**a**) and interleukin-6 levels (**b**) in the two groups. *** means *P* < 0.001. *Pre-OP* Preoperative, *POD* Postoperative day, *POW* Postoperative week, *POM* Postoperative month

In terms of inflammatory markers, the levels of CRP and IL-6 in the TXA group on POD 1 and POD 2, and at POW 1 and POW 2 were significantly lower than those in the control group (*P* < 0.001 for all), whereas no significant difference was observed at POM 1, POM 3 or POM 6 (Fig. 2).

**Fig. 3:**
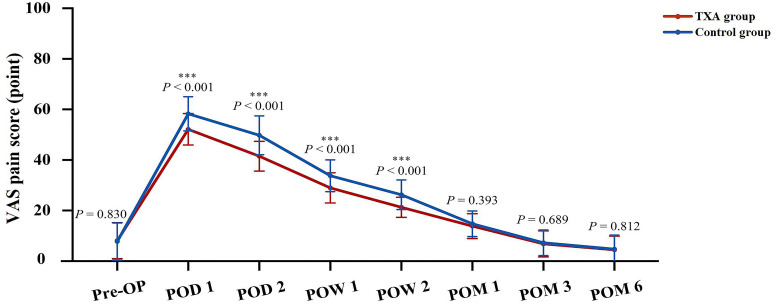
Perioperative VAS pain score between the two groups. *** means *P* < 0.001. *VAS*, Visual analog scale, *Pre-OP* Preoperative, *POD* Postoperative day, *POW* Postoperative week, *POM* Postoperative month

The postoperative VAS pain scores in the TXA group on POD 1 and POD 2, and at POW 1 and POW 2 were significantly lower than those in the control group (*P* < 0.001 for all). However, there was no significant difference in the VAS pain scores between the two groups at POM 1, POM 3 or POM 6 (Fig. 3).

**Fig. 4:**
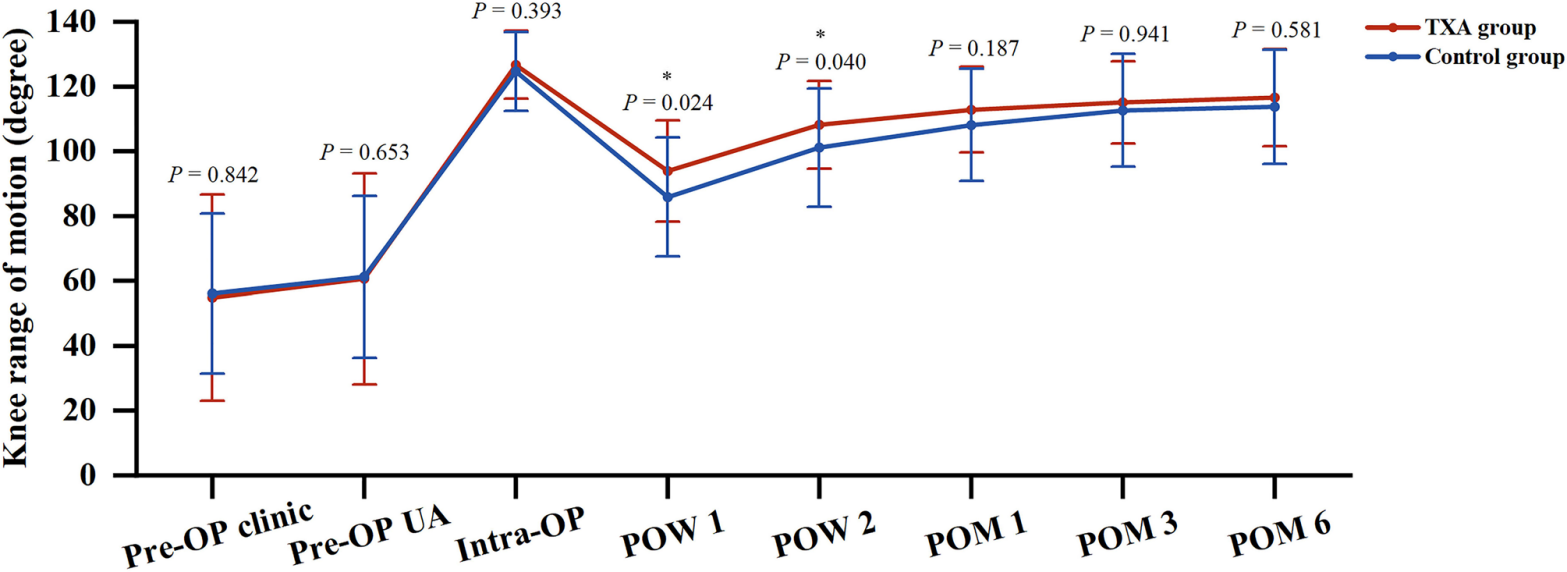
Perioperative knee range of motion between the two groups. * means *P* < 0.05. *Pre-OP* Preoperative, *UA* Under anesthesia, *Intra-OP* Intraoperative, *POW* Postoperative week, *POM* Postoperative month

The TXA group showed significantly better postoperative ROM at POW 1 and POW 2 compared with the control group (*P* = 0.024 and *P* = 0.040, respectively), but no significant difference was found at POM 1, POM 3 or POM 6 between the two groups (Fig. 4). Furthermore, the two groups were comparable in the loss of postoperative ROM (10.11°±6.88° vs. 10.87°±8.54°, *P* = 0.819) and the improvement of postoperative ROM (61.70°±25.48° vs. 57.63°±17.32°, *P* = 0.797) at POM 6.

**Fig. 5:**
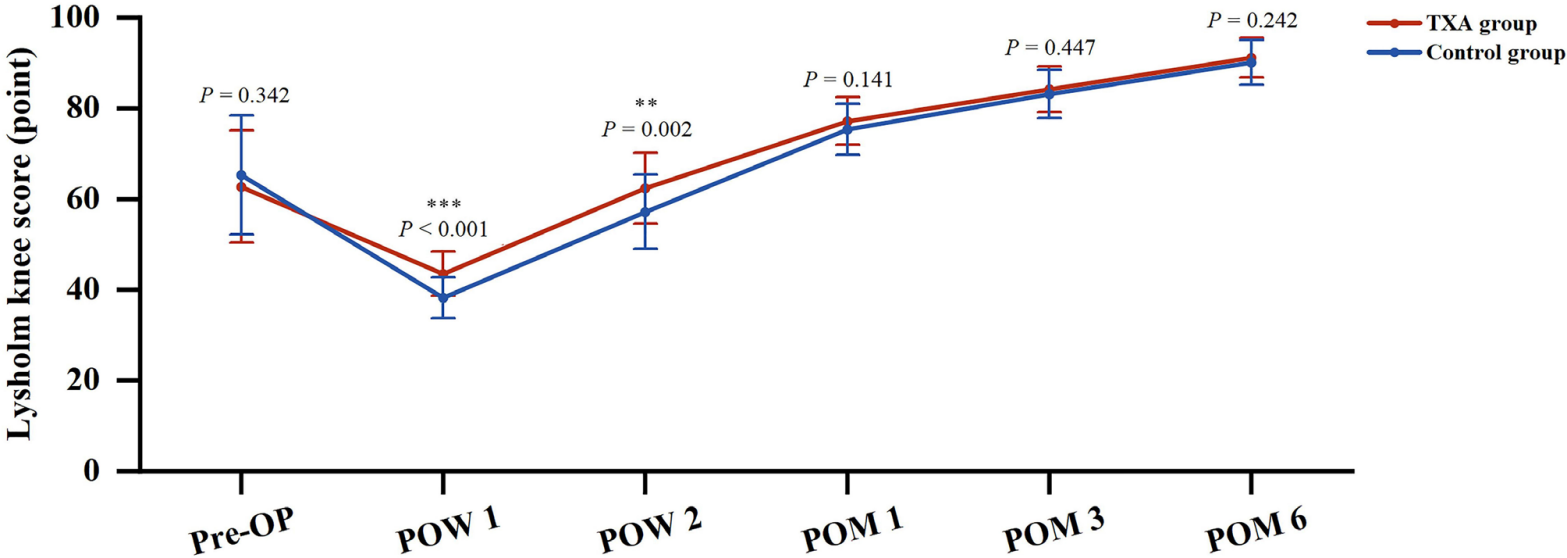
Perioperative Lysholm knee score between the two groups. ** means *P* < 0.01, *** means *P* < 0.001. *Pre-OP* Preoperative, *POW* Postoperative week, *POM* Postoperative month

Similarly, the postoperative Lysholm knee scores in the TXA group were significantly higher than those in the control group at POW 1 and POW 2 (*P* < 0.001 and *P* = 0.002, respectively). There was no significant difference in the Lysholm knee scores between the two groups at POM 1, POM 3 or POM 6 (Fig. 5).

The excellent and good rate was 93.62% in the TXA group and 92.50% in the control group at the sixth postoperative month (Table 3). No significant difference in the excellent and good rate was found between the two groups (χ^2^ = 0.382, *P* = 0.536).

**Table 3:** The excellent and good rates in the TXA group and the control group

	TXA group (n = 47)	Control group (n = 40)
Excellent (n)	41	33
Good (n)	3	4
Fair (n)	3	3
Poor (n)	0	0
Excellent and good rate (%)	93.62	92.50

All the wounds healed by first intention in both groups. Two patients in the control group reported recurrent hemarthrosis after extubation and then underwent aspiration in the clinic. There was no complication such as DVT, infection, fracture, knee instability, or neurovascular injury in any of the patients.

## Discussion

The main findings of this study were that topical administration of TXA in knee arthroscopic arthrolysis could effectively reduce postoperative blood loss and inflammatory response without increased risks. Furthermore, topical administration of TXA alleviated early postoperative pain and improved early postoperative knee ROM and function in patients with knee arthrofibrosis.

Knee arthrofibrosis is a serious complication of knee injury, surgery, and infection caused by massive intra-articular proliferation of fibroblasts and increased synthesis of extracellular matrix proteins [22]. The consequential hyperplastic scars of knee arthrofibrosis can lead to pain and loss of ROM, which is characterized by the clinical features of inflammation, peripatellar swelling, generalized capsular thickening and the exaggerated fibrotic response with a certain amount of tissue contraction or shrinkage [23]. The present literature shows that aberrant inflammatory-wound healing interactions may be the source of chronic inflammatory and pathological knee arthrofibrosis [2]. Indeed, knee arthrofibrosis is affected by both intra-articular and extra-articular factors. The former includes the tissue remodeling, excessive proliferation of fibrous scar tissue, retraction of peri-articular soft tissue, and bone impingement caused by intra-articular malunion. And the latter includes the quadriceps adhesions to the femoral callus, femoral aponeurosis and intermuscular septum, retraction of the muscle due to scar tissue and skin adhesions in the deeper layers [24].

Current conservative treatment options for knee arthrofibrosis include physiotherapy, serial casting, oral corticosteroids, IL-1 receptor antagonists, and MUA. Studies have shown that conservative treatment such as MUA has been successful in more than half of patients with knee arthrofibrosis, thus avoiding surgical treatment. For patients without significant improvement in knee ROM after conservative treatment, further surgical treatment such as open arthrolysis, quadricepsplasty, mini-incision operation and arthroscopic arthrolysis can be performed [25, 26]. Compared with open surgery, arthroscopic arthrolysis has the advantages of less tissue trauma, less postoperative pain, fewer complications, faster recovery and better outcome. However, arthroscopic arthrolysis is insufficient to treat knee arthrofibrosis caused by a combination of extra-articular factors, which may require additional open surgery technique such as quadricepsplasty [27–29].

Tranexamic acid has been widely used in orthopedic surgery and is highly effective in reducing the risk of perioperative blood loss in total hip and knee arthroplasty [30–32]. In contrast to its main action on bone tissue in orthopedic surgery, the effect of topical TXA remains uncertain in arthroscopic surgery, which mainly targets the intra-articular soft tissue. A few studies have demonstrated that intravenous administration of TXA in patients undergoing ACLR can effectively reduce early postoperative blood loss and alleviate pain [19, 20, 33–35]. Meanwhile, Chiang et al. showed that topical TXA could reduce early postoperative drainage and alleviate pain after ACLR, but there were no significant differences in knee ROM and functional score between the TXA group and the control group at POW 4 [18]. However, Lee et al. reported that intra-articular administration of TXA failed to significantly reduce early postoperative blood loss or pain after ACLR [36]. Unlike the previous studies, it used the Hb-balance method as an indirect method to calculate blood loss instead of using a drainage system. In a recent meta-analysis, evidence has shown that the use of TXA in patients undergoing ACLR can reduce postoperative drainage output and hemarthrosis, and improve pain scores and knee ROM in the initial postoperative period without increased complications [37].

The application of TXA in arthroscopic surgery is not only limited to ACLR. Nugent et al. investigated the short-term benefits of TXA in patients undergoing arthroscopic meniscectomy [17]. Their study showed that intravenous TXA in arthroscopic meniscectomy could improve early functional recovery, but was less effective in reducing postoperative knee swelling and pain scores or improving postoperative knee ROM, which may be related to the characteristics of arthroscopic meniscectomy such as shorter operation time, less tissue trauma and lower blood loss. In hip arthroscopy, Karaaslan et al. found that the administration of TXA by infusion could significantly reduce blood loss with negligible side effects when compared with the administration of TXA by injection [38]. Several studies have also explored the administration of TXA in shoulder arthroscopic surgery. Sun et al. concluded in a recent meta-analysis that TXA did not significantly improve intraoperative visual field clarity or relieve postoperative pain in patients who underwent shoulder arthroscopic surgery despite its reliable safety [39]. However, another meta-analysis by Goldstein et al. including seven randomized controlled trials (RCTs) of knee and shoulder arthroscopic surgery suggested that the use of TXA was significantly effective in increasing visual field clarity and technical ease, improving pain scores within six postoperative weeks, and reducing postoperative drainage output and the incidence of hemarthrosis with a lower need for joint aspirations [40].

In our study, the postoperative drainage volumes in the TXA group were significantly lower than those in the control group, and simultaneously the postoperative hemoglobin and hematocrit levels were also significantly different between the two groups, suggesting that topical TXA could effectively reduce postoperative blood loss in knee arthroscopic arthrolysis. Moreover, we observed that topical TXA decreased the VAS pain scores in the early postoperative period, which helped patients start functional exercise sooner with consequent improvement in early postoperative knee ROM and functional scores. One reason for this was that TXA reduced postoperative intra-articular blood loss and alleviated knee swelling. On the other hand, the lower levels of inflammatory markers in the TXA group within two postoperative weeks confirmed the anti-inflammatory effect of TXA on intra-articular soft tissue, which was beneficial for alleviating postoperative pain and accelerating recovery [41].

Although the VAS pain scores in the TXA group were significantly lower than those in the control group within two postoperative weeks, no significant difference was observed during the subsequent follow-up period. This may result from the decrease in the local inflammatory response during the acute phase and the gradual absorption of residual effusion in the joint cavity after the surgery. Correspondingly, we also found no significant differences in knee ROM and functional scores between the two groups beyond POW 2. These results were similar to the findings of a study by Zhang et al. investigating the topical use of TXA in elbow joint open arthrolysis [12]. Furthermore, the postoperative knee ROM was decreased in both groups at follow-up when compared with the intraoperative ROM, which was considered to be related to insufficient postoperative rehabilitation and recurrent arthrofibrosis [3]. Meanwhile, our study showed that topical TXA failed to reduce the loss of postoperative ROM or improve the curative effect in knee arthroscopic arthrolysis at the end of the six-month follow-up period.

Previous studies have evaluated the efficacy and safety of TXA using different administration methods. In orthopedic surgery, intravenous and topical TXA are the two most commonly used routes of administration. Evidence has shown that compared with intravenous TXA, topical TXA has similar efficacy and safety in reducing blood loss without increased risk of thromboembolic complications [42–46]. In our study, no patient in the TXA group experienced complications and adverse events during the six-month follow-up period, which demonstrated the safety of topical TXA in arthroscopic surgery to a certain extent. In effect, few clinical studies have reported the negative effects of topical TXA on the intra-articular tissue, even though its efficacy and safety have been confirmed. The basic researches showed that when the topical TXA concentration exceeded 20 mg/mL, it would produce a time- and dose-dependent cytotoxicity to cartilage, tendon and synovial tissue, which was assumed to result from a caspase-3-dependent apoptotic mechanism [47–52]. Thus, topical administration of TXA at a concentration of 10 mg/mL in the present study was relatively safe.

There were several strengths and limitations in this study. The greatest strength of this study was the first report on the use of TXA in knee arthroscopic arthrolysis, which is one of the procedures with excessive bleeding and extensive intra-articular trauma in arthroscopic surgery. And compared with previous studies on the potential anti-inflammatory effect of TXA, we had a longer-term follow-up of six months for inflammatory marker levels. On the other hand, the main limitation of this study was that it was a single-center retrospective study, so further prospective RCTs with larger sample sizes are necessary to validate our results. Second, this study did not compare the efficacy and safety of different routes of TXA administration in knee arthroscopic arthrolysis, particularly the differences between intravenous and topical TXA. Third, the optimal dose of topical TXA was not determined in arthroscopic arthrolysis, as the topical use of excessive solution may aggravate postoperative knee joint swelling. Additionally, a long-term follow-up is necessary given the potentially time- and dose-dependent cytotoxicity of topical TXA to intra-articular soft tissue.

## Conclusions

Topical administration of TXA in knee arthroscopic arthrolysis can reduce postoperative blood loss and inflammatory response, alleviate early postoperative pain, increase early postoperative knee ROM, and improve early postoperative knee function without increased risks.

## Data Availability

The datasets generated and/or analyzed in the current study are not publicly available because the participants did not consent to release of their data, but they are available from the corresponding author on reasonable request.

## Abbreviations

ROM: Range of motion
ACLR: Anterior cruciate ligament reconstruction
MUA: Manipulation under anesthesia
TXA: Tranexamic acid
BMI: Body mass index
DVT: Deep venous thrombosis
POD: Postoperative day
Hb: Hemoglobin
Hct: Hematocrit
CRP: C-reactive protein
IL-6: Interleukin-6
POW: Postoperative week
POM: Postoperative month
VAS: Visual analog scale
