# Correction: Topical administration of tranexamic acid reduces postoperative blood loss and inflammatory response in knee arthroscopic arthrolysis: a retrospective comparative study

**DOI:** 10.1186/s12891-023-06463-1

**Published:** 2023-05-05

**Authors:** Junqiao Li, Mingke You, Lei Yao, Weili Fu, Qi Li, Gang Chen, Xin Tang, Jian Li, Yan Xiong

**Affiliations:** Department of Orthopedics, Orthopedic Research Institute, West China Hospital, Sichuan University, Chengdu, 610041 China

**Correction:** **BMC Musculoskelet Disord 24, 269 (2023)**

**https://doi.org/10.1186/s12891-023-06349-2**

Following publication of the original article [1], the authors identified an error in the last column of Table 2 (data of P-values were missing some symbols ( <)). The correct table is given below.

**Table 2:** Postoperative drainage volumes and hematologic levels in the two groups

Variables	TXA group (n = 47)	Control group (n = 40)	*P*-value
Drainage volume (mL)			
POD 1	69.26 ± 35.93	153.88 ± 64.02	< 0.001
POD 2	36.49 ± 28.85	94.38 ± 48.39	< 0.001
Total	113.19 ± 69.62	284.75 ± 129.35	< 0.001
Hb (g/L)			
POD 1	140.15 ± 12.79	134.73 ± 9.49	0.030
Extubation	137.04 ± 12.54	128.08 ± 9.83	< 0.001
Loss	10.79 ± 4.32	22.48 ± 6.02	< 0.001
Hct (%)			
POD 1	41.19 ± 3.35	37.93 ± 3.24	< 0.001
Extubation	40.06 ± 3.60	35.38 ± 3.06	< 0.001
Loss	4.15 ± 1.76	9.48 ± 2.10	< 0.001

*POD* Postoperative day, *Hb* Hemoglobin, *Hct* Hematocrit, *CRP* C-reactive protein, *IL-6* interleukin-6

The original article [1] has been corrected.
